# Obesity and Body Composition in Preschool Children with Different Levels of Actigraphy-Derived Physical Activity—A Cross-Sectional Study

**DOI:** 10.3390/jcm9041210

**Published:** 2020-04-23

**Authors:** Justyna Wyszyńska, Piotr Matłosz, Agnieszka Szybisty, Paweł Lenik, Katarzyna Dereń, Artur Mazur, Jarosław Herbert

**Affiliations:** 1 Institute of Health Sciences, Medical College of Rzeszow University, 35-959 Rzeszów, Poland; kderen@ur.edu.pl; 2 Centre for Innovative Research in Medical and Natural Sciences, University of Rzeszow, 35-310 Rzeszów, Poland; 3 Institute of Physical Culture Sciences, Medical College of Rzeszow University, 35-959 Rzeszów, Poland; piotr.matlosz@gmail.com (P.M.); agnieszkaszybisty@wp.pl (A.S.); julialeni66@gmail.com (P.L.); jherbert@ur.edu.pl (J.H.); 4 Institute of Medical Sciences, Medical College of Rzeszow University, 35-959 Rzeszów, Poland; drmazur@poczta.onet.pl

**Keywords:** accelerometer, bioelectrical impedance analysis, obesity, physical activity, preschool children, recommendation, steps

## Abstract

Detailed associations between physical activity (PA) and body composition in preschoolers remain unclear. The aim of this study was to assess body composition among preschool children differentiated according to their levels of PA and to assess whether meeting the current PA recommendations is associated with a lower risk of obesity, determined by body mass index (BMI) and body fat percentage (BFP). Free-living PA was measured using accelerometers for 7 days in children aged 5 to 6 years. Bioelectrical impedance analysis was used to estimate body composition. Significant differences in content of BFP, fat-free mass (FFM), and total body water (TBW) were found between boys meeting and not meeting moderate-to-vigorous PA (MVPA) recommendations. Meeting the MVPA recommendation was associated with a twofold lower risk of obesity determined by BFP in boys but not in girls. In contrast, the total number of recommended steps per day was not related to adiposity in boys or girls. No statistically significant differences were observed in body composition indices and quintiles of MVPA. Boys in the 3rd–5th quintiles of steps per day presented significantly lower BFP and higher muscle mass and TBW than their peers in quintile 1. However, different associations were observed between body composition indices and quintiles of PA.

## 1. Introduction

Overweight and obesity in childhood is one of the most serious public health challenges of the 21st century, having multiple adverse health consequences [1]. Over 41 million children under 5 years of age are estimated to have excess body mass worldwide [2]. In Poland in 2016, the overall prevalence of overweight and obesity in children aged 5 to 6 years was 19.4% and 6.9%, respectively according to World Health Organization (WHO) definitions [3]. It is expected that a considerable fraction of these children with excessive body mass will become obese adults [4]. Therefore, early childhood is an important time frame that should be targeted by preventive strategies intended to reduce body fat, and consequently, decrease the risk of noncommunicable diseases. According to Evensen et al. preventive treatment initiatives among children at high risk of overweight and obesity should start before 5–7 years of age [5]. 

Although obesity is a multifactorial disease, it mainly results from an imbalance between energy intake and energy expenditure. The most modifiable component of energy expenditure is physical activity (PA), being a substantial factor affecting the energy balance equation [6]. Increasing evidence suggests that declining levels of PA are a major factor for the higher prevalence of overfat children [7]. Therefore, the WHO and most public health authorities around the world recommend that children aged 5–17 years should accumulate at least 60 min of moderate-to-vigorous PA (MVPA) daily [8]. In most public health guidelines worldwide, the minimum recommendation of 60 min of MVPA is associated with 10,000–14,000 free-living steps/day in preschool children (approximately aged from 4 to 6 years of age) [9]. Although there is substantial evidence that PA provides significant health benefits to young people, in most European countries, less than 50% of children and adolescents meet these recommendations [10].

The literature shows that there is an inverse relationship between the level of PA and overweight status in school-age children [11]. Available data are often based on the determination of obesity using the body mass index (BMI). However, individuals with increased BMI are not always obese in terms of body composition [12], and BMI is not a direct measure of body fat [13]. The studies also show that individuals can have normal body weight and at the same time excess body fat [14]. A reliable and accurate method for measuring fat content and other body components is bioelectrical impedance analysis (BIA). In addition, this method is fast, non-invasive, and relatively inexpensive [15]. Validation of a BIA device against dual-energy X-ray absorptiometry showed strong significant correlations between both methods for measuring fat mass, body fat percentage (BFP), and total fat-free mass (FFM) [16].

Relatively few studies have examined the association between objectively measured levels of PA and obesity status in preschool children [17,18,19] and, to the best of our knowledge, no studies have compared the compliance of daily PA recommendations and obesity status measured by BFP in 5- to 6-year-old children. Detailed associations between physical activity and body composition in preschoolers remain unclear. Moreover, there are no reports on the comprehensive analysis of actigraphy-based PA measurement with BIA-based body composition, considering different levels of PA among a large sample of preschool children aged 5 to 6 years. Most evidence concerns associations between habitual PA and adiposity. 

Therefore, the aim of this study was to assess body composition among preschool children differentiated according to the levels of objectively measured PA and to assess whether meeting the current PA recommendations is associated with lower risk of obesity as determined by BMI and BFP.

## 2. Materials and Methods

### 2.1. Study Design and Study Sample

A cross-sectional study was conducted at 22 randomly selected kindergartens in Rzeszów, Poland. The study included healthy preschoolers aged 5 to 6 years. Participation in the study was voluntary and anonymous. Anthropometric measurements were taken between 8:00 a.m. and 10:00 a.m. by experienced researchers. Data were collected when preschoolers were attending kindergarten and excluded major holidays.

An invitation to participate in the study was sent to all parents of children aged between 5 to 6 years attending kindergarten. The consent of 707 parents was obtained for child participation in the measurements for the purpose of this study. Of those respondents, 31 were excluded from the study for the following reasons: a functional state that does not allow for self-maintenance of a standing position (*n* = 1), strong anxiety of examination (*n* = 3), taking medication affecting body composition (*n* = 3), a failure to return or complete the survey (*n* = 18), or lack of valid accelerometer data (*n* = 6). Ultimately, the study group consisted of 676 students (51% girls).

### 2.2. Anthropometric Measurements

Anthropometric measurements (body mass, height) were performed under standard conditions. Body height was measured in an upright position, barefoot, to the nearest 0.1 cm using a portable stadiometer (Tanita HR-200, Tokyo, Japan). Body mass was assessed with an accuracy of 0.01 kg using a body composition analyzer (BC-420 MA, Tanita, Tokyo, Japan). The participants were measured in light clothing and wearing no shoes. Body mass index was calculated as body mass/height (kg/m^2^). Based on BMI values, the BMI percentile of individual participants was calculated. Polish BMI percentile charts specific for age, sex, and body height were used [20]. Based on the BMI percentile values, two groups of participants were distinguished: (1) No obesity: <95th percentile of BMI, and (2) Obesity: ≥95th percentile of BMI [21].

### 2.3. Body Composition

Foot-to-foot BIA was used to estimate body composition with a body composition analyzer (BC-420, Tanita). The foot-to-foot method of BIA is a reliable and accurate tool for the measurement of body composition in the pediatric population, however the BIA-BFP overestimated dual-energy X-ray absorptiometry (DEXA) BFP by a mean of 2.53% (limits of agreement are 4.29% and 9.36%) [15]. BIA monitors have been validated against dual-energy X-ray absorptiometry (DEXA) in mixed populations. Results showed strong significant correlations between both methods for fat mass, BFP, and total FFM [16]. BIA was performed in the early morning. Parents of participants were instructed about the necessity of overnight fast before examination (for at least 8 h), because food or beverage consumption may decrease impedance by 4–15 Ω over a 2–4 h period after meals, representing an error smaller than 3% [22]. Associations between body composition and PA levels were assessed. Additionally, two groups of participants were distinguished based on BFP content: (1) No obesity: <95th percentile of BFP; (2) Obesity: ≥95th percentile of BFP. For this purpose, BFP centile values by age and sex were used [23]. 

### 2.4. Physical Activity

Level of PA was objectively monitored using the tri-axial accelerometer (GT3X-BT Monitor; ActiGraph, Pensacola, FL, USA) and was analyzed using the ActiLife 6.13 data analysis software. The device detects body movement acceleration in three planes. ActiGraph accelerometers are a widely used tool to objectively measure PA level in preschool children [24]. The device was attached to the participant’s right hip. Children and parents/ caregivers received detailed instructions on the use of the accelerometer. They were instructed to wear the monitor for 24 h daily for seven consecutive days and nights during all activities, except for water-related activities. In addition, they were asked to maintain their PA patterns. Accelerometer data were collected from October to November each year from 2018 to 2019.

As the accelerometer was worn for 24 h per day, it was necessary to identify nocturnal sleep episode time distinctly, and this was done using a published automated algorithm [25]. After exclusion of the nocturnal sleep episode time, non-wear time was determined as 60 min of consecutive zeros allowing for 2 min of non-zero interruptions [26]. Once nocturnal sleep episode time and non-wear time were computed, waking wear time and different PA levels were calculated and identified using 5 s epoch data. The findings suggested that using 5 s epochs is better adapted to preschool PA patterns [27]. A waking wear time of ≥500 min/day was used as the criterion for a valid day, and ≥4 days were used as the criteria for a valid 7 day period of accumulated data (including ≥3 valid weekdays and ≥1 valid weekend day) [28]. For each participant, the mean MVPA (min/day) and the mean daily step count were calculated. The cutoff points from Evenson et al. were selected to determine the time spent on MVPA [29]. MVPA time was calculated as mean daily minutes ≥2296 counts/min from all valid days. Daily step count was calculated as the mean daily step count from all valid days. Participants with at least 60 min of MVPA per day were considered to have met the PA guidelines, and those with daily MVPA < 60 min were considered to have not met PA guidelines [8]. Regarding the number of daily steps, participants with at least 12,000 steps per day were considered to be sufficiently physically active [30].

The majority of children (*n* = 741, 77.8%) had complete 7 day actigraphic data; 170 (17.8%) and 42 (4.4%) children had 6 and 5 day actigraphic data, respectively. Average monitoring included 6.7 days of recordings.

### 2.5. Statistical Analysis

Statistical analysis was performed using SPSS 20 software (IBM, North Harbour, UK). The data were presented as the mean ± standard deviation (SD) for continuous variables. The normality was tested by the Kolmogorov–Smirnov test. None of the analyzed variables showed compliance with a normal distribution, therefore non-parametric tests were applied. Therefore, the Mann–Whitney U test was used. The odds ratio (OR) with a 95% confidence interval (95% CI) was calculated using logistic regression. To assess the dose–response relationship between PA and body composition indices, participants were divided into 5 groups according to the MVPA and steps per day quintiles. To investigate differences among PA quintiles the Kruskal–Wallis test was used. The level of statistical significance was adopted at *p* < 0.05. 

### 2.6. Ethics

Written informed consent was obtained from parents or legal guardians and participating children prior to participation in the study. The study was approved by the Bioethics Committee at the Medical Department of the University of Rzeszów, decision no. 2018/01/05 on 11 January 2018, and it was conducted in accordance with ethical standards laid down in an appropriate version of the Declaration of Helsinki.

## 3. Results

The final sample consisted of 676 preschoolers aged 5 to 6 years (mean age 5.55 years; 49% male). Mean height was 117.21 ± 6.06 cm and mean weight 21.67 ± 3.89 kg. No significant differences were found with regard to body composition between girls and boys. Mean MVPA was significantly lower in girls than in boys (*p* < 0.001) (Table 1). 

Table 2 shows body composition distribution among participants with different levels of MVPA. Twenty-four percent (24.3%; *n* = 164) of the children accumulated 60 min/day MVPA as recommended by WHO for children. Children with higher levels of MVPA had lower BFP content (*p* = 0.005), and higher content of muscle mass, FFM, and TBW (*p* = 0.026, 0.034, and 0.015, respectively). A higher level of MVPA among boys was significantly associated with a lower content of BFP (*p* = 0.001) and higher content of FFM and TBW (*p* = 0.022 and 0.021, respectively). There were no statistically significant differences in body composition among girls with different levels of MVPA.

Table 3 shows body composition distribution among children with different levels of PA measured by the number of steps per day. Higher muscle mass content was found in the total sample and in boys with greater number of daily steps (*p* < 0.001 and *p* = 0.001, respectively).

Table 4 presents odds ratios for the prevalence of obesity, determined according to BMI or BFP, depending on the MVPA level. It was found that boys who met the WHO recommendation regarding daily MVPA level had a twofold lower risk of obesity categorized based on BFP, than their peers who did not meet the WHO recommendation.

Table 5 presents odds ratios for the prevalence of obesity categorized according to BMI or BFP depending on the daily number of steps. No significant associations were found between the occurrence of obesity, determined by both BMI or BFP, and the level of PA assessed by the number of steps per day.

There were no statistically significant differences in body composition indices between quintiles of MVPA (Table 6).

In boys, BFP showed a significant dose-related trend through the steps per day quintiles, indicating a significant negative correlation between steps per day and BFP. Boys in the 3rd–5th quintiles of steps per day presented higher muscle mass and TBW than boys in the first quintile (Table 7).

## 4. Discussion

To our knowledge, this is the first study to examine comprehensive associations between body composition and objectively measured levels of PA in a large sample of preschool children aged from 5 to 6 years. The present results showed that in the total sample of preschoolers aged from 5 to 6 years, a level of MVPA below the WHO recommendation was significantly associated with higher content of body fat by 1.18% and lower content of muscle mass, FFM, and TBW by 0.8%, 1.19%, and 0.64%, respectively. When analyzing the above associations according to gender, it was found that these relationships were more apparent in boys. Body fat content in boys, who accumulated at least 60 min of MVPA daily, was significantly lower by 1.62% than in their less-active peers. In girls, these relationships occurred but were not statistically significant. However, different associations were observed when body composition indices were analyzed with quintiles of PA. No statistically significant differences were observed in body composition parameters and dose-related MVPA. Boys in the 3rd–5th quintiles of steps per day presented significantly lower BFP and higher muscle mass and TBW than their peers in quintile 1.

There were no clear associations between body composition and levels of PA evaluated by number of daily steps. The mean value of muscle mass content was higher by 2.28% in boys with at least 12,000 steps per day. It was also found that boys who met recommendation of minimal level of MVPA per day had a twofold lower risk of obesity determined by BFP. However, these associations were not observed in participants who met the recommendation regarding number of steps per day. An opposite association was found by Vale et al., who showed that overweight and obese girls, not boys, were approximately four times more likely to not meet the minimum 60 mins of daily MVPA than their non-overweight counterparts [17]. This difference may result from, among others, discrepancies in the methods used herein and in other studies (body status evaluated by BMI and cutoff point for MVPA was ≥1680 counts/min). In a study by Diouf et al., 90% of the children aged 8–11 years, who accumulated at least 60 min/day of MVPA were of normal or underweight status as categorized by BMI values. A small proportion of overweight/obese children met that recommendation [31]. Similar results have been reported from previous studies on school age children, indicating that overweight children are significantly less likely to practice PA than their normal weight and underweight peers [32,33,34]. In middle-school girls, the odds of being overweight or overfat were substantially increased among girls who had low levels of physical activity (15th percentile) in comparison with girls who were active (85th percentile) (OR ranged from 1.59 to 3.82) [35].

Regarding MVPA levels in association with muscle mass, FFM, and TBW, the present results cannot be compared to those of similar studies. To the best of our knowledge, the literature contains no such reports. A study conducted among a group of 180 adolescents showed no association between PA variables and FFM, or between meeting/not meeting PA recommendations and FFM in adolescents studied [36]. Similar results were presented in study by Vicente-Rodriguez et al., who showed that meeting PA recommendations was not associated with higher levels of FFM, in prepubertal girls, which may indicate that other type of stimulus (e.g., regular sport participation) is needed in order to improve FFM [37]. Morelli et al. showed that adolescents performing vigorous-intensity PA have better body composition parameters reflecting lean metabolic active body mass [38]. The results of another study suggested that fifteen minutes of accumulated vigorous PA per day is associated with 0.36 lower percentage of body fat in children aged 4 years [39].

Physical activity has been considered a powerful marker of health in children and adolescents. However, the reported level of PA of children has decreased during the last decades [40]. A low level of PA is a significant and potentially modifiable cause of obesity in the pediatric population. An inverse cross-sectional association exists between MVPA and child adiposity [11]. Therefore, MVPA is stated as an important factor of pediatric obesity prevention, and at least 60 min MVPA per day is recommended to ensure childhood health [8]. Our findings for boys support the hypothesis that higher levels of habitual PA might have a protective role against higher levels of adiposity, even at young ages. The risk of obesity assessed by BFP was twofold higher in boys with MVPA below 60 min per day, than in peers who accumulated MVPA of least 60 min. However, there were no significant associations between the level of MVPA and prevalence of obesity assessed by BMI both in boys and girls. Moreover, no significant associations were found in the occurrence of obesity in the study population with different levels of PA assessed by the number of steps. The trend of obesity being less frequent in the total sample of children with at least 12,000 steps per day is noted, but this association is not statistically significant. This confirms that the main disadvantage of assessment of the level of PA by the number of steps is the inability to assess the intensity of PA, and consequently an inaccurate estimation of energy expenditure [41].

Due to our study design (cross-sectional), it is not possible to infer a causal relationship of PA with body composition. Moreover, we cannot be certain that confounders such as nutrition or genetic factors have not influenced our observations. Although accurate measures of energy intake would have enriched our ability to interpret the results, lack of those measures does not negate the relevance of this study to public health. The aim here was to show the associations between levels of MVPA with body composition in a free-living population of preschool children. The next limitation of the study was a single measurement of body height, thus intra-observer variability could not be calculated. However, body height measurement was always performed by the same researcher and there was no inter-observer variability between measurements. Another limitation of this study was not adjusting fat mass to an unrelated measure of body fat such as height that could improve interpretation when assessing adiposity [42]. In children, the fat mass index was demonstrated to better discriminate adiposity than the BFP [43,44]. Another limitation of the study was the fact that the accuracy of muscle mass (%) measurement by the device used in this study was unknown, and the socioeconomic status of the parents of the preschoolers was not included in the analyzes. Therefore, outcomes related to muscle mass should be interpreted with caution.

It is possible that associations between PA and body composition would have been stronger if energy intake had been controlled. The study, however, benefits from a number of strengths: a relatively large sample size, a focus on PA levels in preschool children, and an assessment of compliance with PA recommendations using an objective PA measure. The 5 s epoch used in this study appears to capture a greater amount of data in preschool children. Moreover, body composition was measured by using the BIA method that has demonstrated excellent test–retest reliability, moderately strong absolute agreement with DEXA, and high specificity for overfat and obese classification. Compared to DEXA, BIA is a portable and efficient means of assessing BFP in school children [22]. 

Future research should include a greater emphasis on longitudinal studies to examine how objectively measured PA and body composition vary by age within an individual over time, taking into account possible confounding factors. Multiple longitudinal cohorts with different ages of participants would help to corroborate and extend our findings. Further research should also seek underlying mechanisms.

## 5. Conclusions

Among boys, failure to meet the daily recommendation for MVPA was associated with a higher risk of obesity categorized by BFP, as well as with a significant difference in content of BFP, FFM, and TBW. In contrast, the total number of recommended steps per day was not related to adiposity in both boys and girls. However, different associations were observed between body composition indices and levels of PA by quintiles. Prospective study designs are needed to determine the direction of the association between PA intensity and body composition in early childhood.

## Figures and Tables

**Table 1:** Characteristics of the study population.

Variable	All Subjects (*n* = 676)		Boys (*n* = 331)		Girls (*n* = 345)		*p*
	Mean	SD	Mean	SD	Mean	SD	
Age (years)	5.55	0.50	5.55	0.50	5.56	0.50	0.742
Body height (cm)	117.2	6.1	117.9	6.1	116.5	5.9	**0.006**
Body mass (kg)	21.67	3.89	22.09	4.13	21.26	3.61	**0.008**
BMI	15.68	1.76	15.78	1.81	15.57	1.70	0.147
BFP (%)	20.36	4.73	20.33	4.68	20.38	4.79	0.291
Muscle mass (%)	76.65	4.66	76.52	4.35	76.77	4.94	0.619
FFM (%)	79.64	4.73	79.67	4.67	79.62	4.77	0.173
TBW (%)	58.40	3.19	58.49	3.03	58.31	3.34	0.201
MVPA day (min)	47.53	22.47	51.01	23.91	44.20	20.48	**<0.001**
Steps/day	9182.66	2322.82	9374.47	2548.14	8998.64	2070.99	**0.034**

*p*-value represents the differences between boys and girls; BFP—body fat percentage; BMI—body mass index; FFM—fat-free mass; MVPA—moderate-to-vigorous physical activity; TBW—total body water; significant associations are highlighted in bold.

**Table 2:** Body composition among children with different levels of moderate-to-vigorous physical activity.

Variable	MVPA/Day						*p*
	<60 min			≥60 min			
	Mean	SD	Me	Mean	SD	Me	
**All Subjects**							
	(*n* = 512)			(*n* = 164)			
BFP (%)	20.64	4.99	19.80	19.46	3.69	18.85	**0.005**
Muscle mass (%)	76.45	4.85	76.59	77.25	3.93	77.45	**0.026**
FFM (%)	79.35	4.98	80.2	80.54	3.67	81.15	**0.034**
TBW (%)	58.24	3.33	58.73	58.88	2.67	59.30	**0.015**
**Girls**							
	(*n* = 278)			(*n* = 67)			
BFP (%)	20.51	4.92	20.05	19.86	4.16	19.00	0.241
Muscle mass (%)	76.65	5.07	76.72	77.29	4.32	77.84	0.186
FFM (%)	79.49	4.91	79.95	80.14	4.12	81.00	0.673
TBW (%)	58.25	3.43	58.51	58.55	2.97	59.10	0.366
**Boys**							
	(*n* = 234)			(*n* = 97)			
BFP (%)	20.80	5.08	19.55	19.18	3.32	18.60	**0.001**
Muscle mass (%)	76.23	4.58	76.50	77.22	3.66	77.14	0.058
FFM (%)	79.19	5.06	80.45	80.82	3.29	81.40	**0.022**
TBW (%)	58.23	3.21	58.80	59.11	2.43	59.36	**0.021**

BFP—body fat percentage; FFM—fat-free mass; Me—median; MVPA—moderate-to-vigorous physical activity; TBW—total body water; significant associations are highlighted in bold.

**Table 3:** Body composition among children with different numbers of steps per day.

Variable	Steps/Day						*p*
	<12,000			≥12,000			
	Mean	SD	Me	Mean	SD	Me	
**All Subjects**							
	(*n* = 601)			(*n* = 75)			
BFP (%)	20.46	4.83	19.60	19.52	3.75	18.80	0.103
Muscle mass (%)	76.45	4.70	76.65	78.23	3.94	78.46	**<0.001**
FFM (%)	79.54	4.83	80.40	80.48	3.72	81.20	0.294
TBW (%)	58.34	3.25	58.80	58.84	2.69	59.16	0.209
**Girls**							
	(*n* = 316)			(*n* = 29)			
BFP (%)	20.39	4.85	20.00	20.24	4.10	19.10	0.844
Muscle mass (%)	76.68	4.99	76.86	77.82	4.28	78.80	0.113
FFM (%)	79.61	4.81	80.00	79.76	4.02	80.90	0.111
TBW (%)	58.31	3.39	58.64	58.22	2.87	58.70	0.800
**Boys**							
	(*n* = 285)			(*n* = 46)			
BFP (%)	20.53	4.83	19.40	19.07	3.48	18.50	0.051
Muscle mass (%)	76.20	4.36	76.47	78.48	3.73	78.28	**0.001**
FFM (%)	79.47	4.81	80.60	80.93	3.43	81.50	0.879
TBW (%)	58.37	3.09	58.97	59.22	2.53	59.63	0.067

BFP—body fat percentage; FFM—fat-free mass; Me—median; TBW—total body water; significant associations are highlighted in bold.

**Table 4:** Multinomial regression analysis showing the association between the MVPA recommendation and obesity status.

Body Mass Category		MVPA/Day				OR (95% CI)	*p*
		<60 min		≥60 min			
		*n*	%	*n*	%		
**All subjects**							
BMI	No obesity	488	95.3	158	96.3	0.77 (0.31–1.92)	0.578
	Obesity	24	4.7	6	3.7		
BFP	No obesity	406	79.3	141	86.0	0.62 (0.38–1.02)	0.058
	Obesity	106	20.7	23	14.0		
**Girls**							
BMI	No obesity	267	96.0	64	95.5	1.14 (0.31–4.20)	0.846
	Obesity	11	4.0	3	4.5		
BFP	No obesity	235	84.5	59	88.1	0.54 (0.15–1.95)	0.342
	Obesity	43	15.5	8	11.9		
**Boys**							
BMI	No obesity	221	94.4	94	96.9	0.74 (0.33–1.66)	0.465
	Obesity	13	5.6	3	3.1		
BFP	No obesity	171	73.1	82	84.5	0.50 (0.27–0.92)	**0.025**
	Obesity	63	26.9	15	15.5		

BFP—body fat percentage; BMI—body mass index; MVPA—moderate-to-vigorous physical activity; OR (95% CI)—odds ratio with a 95% confidence interval; significant associations are highlighted in bold.

**Table 5:** Multinomial regression analysis showing the association between the steps per day recommendation and obesity status.

Body Mass Category		Steps/Day				OR (95% CI)	*p*
		<12,000		≥12,000			
		*n*	%	*n*	%		
**All Subjects**							
BMI	No obesity	573	95.3	73	97.3	0.56 (0.13–2.40)	0.43
	Obesity	28	4.7	2	2.7		
BFP	No obesity	481	80.0	66	88.0	0.55 (0.26–1.13)	0.098
	Obesity	120	20.0	9	12.0		
**Girls**							
BMI	No obesity	304	96.2	27	93.1	1.88 (0.40–8.82)	0.418
	Obesity	12	3.8	2	6.9		
BFP	No obesity	268	84.8	26	89.7	0.85 (0.82–0.89)	0.099
	Obesity	48	15.2	3	10.3		
**Boys**							
BMI	No obesity	269	94.4	46	100.0	0.64 (0.19–2.21)	0.482
	Obesity	16	5.6	0	0.0		
BFP	No obesity	213	74.7	40	87.0	0.44 (0.18–1.09)	0.070
	Obesity	72	25.3	6	13.0		

BFP—body fat percentage; BMI—body mass index; OR (95% CI)—odds ratio with a 95% confidence interval.

**Table 6:** Body composition indices according to the quintiles of MVPA.

Variable	MVPA/Day										
	Quintile 1 (<29.29 min)		Quintile 2 (29.29–39.31 min)		Quintile 3 (39.32–49.19 min)		Quintile 4 (49.2–63.29 min)		Quintile 5 (>63.29 min)		*p*
	Mean	SD	Mean	SD	Mean	SD	Mean	SD	Mean	SD	
**All Subjects**											
BMI	15.85	2.19	15.61	**1.76**	15.66	1.59	15.78	1.75	15.48	1.39	0.766
BFP (%)	21.15	5.76	20.56	4.92	20.50	4.58	20.12	4.45	19.43	3.58	0.101
Muscle mass (%)	76.31	5.35	76.38	4.94	76.54	4.41	76.80	4.68	77.21	3.74	0.457
FFM (%)	78.85	5.12	79.44	5.05	79.50	4.84	79.88	4.70	80.57	4.26	0.180
TBW (%)	58.01	3.59	58.23	3.46	58.32	3.07	58.53	3.13	58.90	2.57	0.191
**Girls**											
BMI	15.60	1.92	15.58	1.71	15.70	1.64	15.57	1.65	15.35	1.50	0.756
BFP (%)	20.62	5.69	20.53	4.87	20.46	4.54	20.12	4.17	19.95	4.22	0.787
Muscle mass (%)	77.03	5.65	76.29	5.17	76.88	4.73	76.75	4.47	77.07	4.24	0.804
FFM (%)	79.38	5.03	79.47	5.03	79.54	4.97	79.88	4.51	80.05	4.71	0.848
TBW (%)	58.30	3.67	58.17	3.55	58.23	3.27	58.45	3.03	58.47	2.99	0.859
**Boys**											
BMI	16.20	2.49	15.66	1.87	15.63	1.55	15.95	1.83	15.56	1.31	0.797
BFP (%)	21.87	5.82	20.62	5.06	20.53	4.65	20.12	4.70	19.10	3.09	0.058
Muscle mass (%)	75.31	4.77	76.54	4.54	76.24	4.12	76.84	4.88	77.29	3.41	0.103
FFM (%)	78.13	5.27	79.38	5.15	79.47	4.76	79.88	4.89	80.90	3.91	0.154
TBW (%)	57.62	3.47	58.34	3.32	58.40	2.89	58.59	3.24	59.17	2.25	0.088

BFP—body fat percentage; BMI—body mass index; FFM—fat-free mass; MVPA—moderate-to-vigorous physical activity; TBW—total body water.

**Table 7:** Body composition indices according to the quintiles of steps per day.

Variable	Steps/Day										
	Quintile 1 (<7281.40)		Quintile 2 (7281.40–8468.46)		Quintile 3 (8468.47–9472.28)		Quintile 4 (9472.29–10,926.74)		Quintile 5 (>10,926.74)		*p*
	Mean	SD	Mean	SD	Mean	SD	Mean	SD	Mean	SD	
**All Subjects**											
BMI	16.06	2.09	15.76	1.75	15.56	1.70	15.50	1.65	15.50	1.50	0.116
BFP (%)	21.78	6.14	20.59	4.69	19.96	4.20	19.77	4.10	19.69	3.94	**0.031**
Muscle mass (%)	75.62	5.36	76.09	4.79	76.93	4.31	77.05	4.37	77.55	4.15	**0.002**
FFM (%)	78.22	5.20	79.41	4.73	80.04	4.93	80.23	4.59	80.42	4.57	0.425
TBW (%)	57.72	3.69	58.22	3.25	58.60	3.06	58.74	2.99	58.71	2.83	0.063
**Girls**											
BMI	16.03	2.07	15.49	1.58	15.27	1.12	15.59	1.76	15.48	1.83	0.303
BFP (%)	21.81	5.90	19.97	4.44	19.47	3.73	20.39	4.85	20.32	4.59	0.248
Muscle mass (%)	75.60	5.79	76.72	4.81	77.66	4.20	76.91	4.93	77.05	4.71	0.172
FFM (%)	78.19	5.19	80.03	4.55	80.53	4.18	79.61	5.30	79.68	4.94	0.256
TBW (%)	57.45	3.81	58.58	3.25	58.95	2.71	58.30	3.50	58.20	3.26	0.223
**Boys**											
BMI	16.09	2.13	16.20	1.92	15.84	2.09	15.43	1.55	15.51	1.18	0.124
BFP (%)	21.75	6.43	21.60	4.95	20.43	4.59	19.20	3.19	19.18	3.28	**0.010**
Muscle mass (%)	75.64	4.92	75.05	4.62	76.22	4.33	77.18	3.81	77.94	3.63	**0.002**
FFM (%)	78.25	5.22	78.40	4.90	79.57	5.58	80.80	3.71	80.82	4.24	0.207
TBW (%)	58.00	3.57	57.62	3.18	58.26	3.35	59.15	2.37	59.12	2.38	**0.018**

BFP—body fat percentage; BMI—body mass index; FFM—fat-free mass; TBW—total body water, significant associations are highlighted in bold.

## References

[1] De Onis M., Blössner M., Borghi E. (2010). Global prevalence and trends of overweight and obesity among preschool children. Am. J. Clin. Nutr..

[2] World Health Organization Facts and Figures on Childhood Obesity. https://www.who.int/end-childhood-obesity/facts/en/.

[3] Kułaga Z., Gurzkowska B., Grajda A., Wojtyło M., Góźdź M., Litwin M. (2016). The prevalence of overweight and obesity among polish pre-school-aged children. Dev. Period Med..

[4] Herman K.M., Craig C.L., Gauvin L., Katzmarzyk P.T. (2009). Tracking of obesity and physical activity from childhood to adulthood: The Physical Activity Longitudinal Study. Int. J. Pediatr. Obes..

[5] Evensen E., Wilsgaard T., Furberg A.S., Skeie G. (2016). Tracking of overweight and obesity from early childhood to adolescence in a population-based cohort—the Tromsø Study, Fit Futures. BMC Pediatr..

[6] Swift D.L., Johannsen N.M., Lavie C.J., Earnest C.P., Church T.S. (2014). The role of exercise and physical activity in weight loss and maintenance. Prog. Cardiovasc. Dis..

[7] Ng M., Fleming T., Robinson M., Thomson B., Graetz N., Margono C. (2014). Global, regional, and national prevalence of overweight and obesity in children and adults during 1980–2013: A systematic analysis for the Global Burden of Disease Study 2013. Lancet.

[8] World Health Organization Physical Activity and Young People. Recommended Levels of Physical Activity for Children Aged 5–17 Years. https://www.who.int/dietphysicalactivity/factsheet_young_people/en/.

[9] Tudor-Locke C., Craig C.L., Beets M.W., Belton S., Cardon G.M., Duncan S., Hatano Y., Lubans D.R., Olds T.S., Raustorp A. (2011). How many steps/day are enough? For children and adolescents. Int. J. Behav. Nutr. Phys. Act..

[10] World Health Organization (2018). Physical Activity Factsheets for the 28 European Union Member States of the Who European Region. http://www.euro.who.int/__data/assets/pdf_file/0005/382334/28fs-physical-activity-euro-rep-eng.pdf?ua=1.

[11] Jiménez-Pavón D., Kelly J., Reilly J.J. (2010). Associations between objectively measured habitual physical activity and adiposity in children and adolescents: Systematic review. Int. J. Pediatr. Obes..

[12] Bucyk B., Tupikowska M., Bednarek-Tupikowska G. (2009). Diagnostic criteria for metabolic obesity in normal weight subjects. Endokr Otyłość i Zab Przem Ma.

[13] Pietrobelli A., Tatò L. (2005). Body composition measurements: From the past to the future. Acta Paediatr..

[14] Conus F., Rabasa-Lhoret R., Péronnet F. (2007). Characteristics of metabolically obese normal-weight (MONW) subjects. Appl. Physiol. Nutr. Metab..

[15] Tyrrell V.J., Richards G., Hofman P., Gillies G.F., Robinson E., Cutfield W.S. (2001). Foot-to-foot bioelectrical impedance analysis: A valuable tool for the measurement of body composition in children. Int. J. Obes..

[16] Karelis A.D., Chamberland G., Aubertin-Leheudre M., Duval C. (2013). Validation of a portable bioelectrical impedance analyzer for the assessment of body composition. Appl. Physiol. Nutr. Metabol..

[17] Vale S., Trost S., Ruiz J.J., Rêgo C., Moreira P., Mota J. (2013). Physical activity guidelines and preschooler’s obesity status. Int. J. Obes. (Lond.).

[18] Reilly J.J., Armstrong J., Dorosty A.R., Emmett P.M., Ness A., Rogers I., Steer C., Sherriff A. (2005). Avon Longitudinal Study of Parents and Children Study Team. Early life risk factors for obesity in childhood: Cohort study. BMJ.

[19] Trost S.G., Sirard J.R., Dowda M., Pfeiffer K.A., Pate R.R. (2003). Physical activity in overweight and nonoverweight preschool children. Int. J. Obes. Relat. Metab. Disord..

[20] Kułaga Z., Różdżyńska A., Palczewska I. (2010). Percentile charts of height, body mass and body mass index in children and adolescents in Poland—results of the OLAF study. Stand Med..

[21] Barlow S.E., Expert Committee (2007). Expert committee recommendations regarding the prevention, assessment, and treatment of child and adolescent overweight and obesity: Summary report. Pediatrics.

[22] Kushner R.F., Gudivaka R., Schoeller D.A. (1996). Clinical characteristics influencing bioelectrical impedance analysis measurements. Am. J. Clin. Nutr..

[23] McCarthy H.D., Cole T.J., Fry T., Jebb S.A., Prentice A.M. (2006). Body fat reference curves for children. Int. J. Obes. (Lond.).

[24] Leeger-Aschmann C.S., Schmutz E.A., Zysset A.E., Kakebeeke T.H., Messerli-Bürgy N., Stülb K., Arhab A., Meyer A.H., Munsch S., Jenni O.G. (2019). Accelerometer-derived physical activity estimation in preschoolers—comparison of cut-point sets incorporating the vector magnitude vs the vertical axis. BMC Public Health.

[25] Tudor-Locke C., Barreira T.V., Schuna J.M., Mire E.F., Katzmarzyk P.T. (2014). Fully automated waist-worn accelerometer algorithm for detecting children’s sleep-period time separate from 24-h physical activity or sedentary behaviors. Appl. Physiol. Nutr. Metab..

[26] Troiano R.P., Berrigan D., Dodd K.W., Mâsse L.C., Tilert T., McDowell M. (2008). Physical activity in the United States measured by accelerometer. Med. Sci. Sports Exerc..

[27] Vale S., Santos R., Silva P., Soares-Miranda L., Mota J. (2009). Preschool children physical activity measurement: Importance of epoch length choice. Pediatr. Exerc. Sci..

[28] Hinkley T., O’Connell E., Okely A.D., Crawford D., Hesketh K., Salmon J. (2012). Assessing volume of accelerometry data for reliability in preschool children. Med. Sci. Sports Exerc..

[29] Evenson K.R., Catellier D.J., Gill K., Ondrak K.S., McMurray R.G. (2008). Calibration of two objective measures of physical activity for children. J. Sports Sci..

[30] World Health Organization (2005). WHO STEPS Surveillance Manual: The WHO STEP Wise Approach to Chronic Disease Risk Factor Surveillance.

[31] Diouf A., Thiam M., Idohou-Dossou N., Diongue O., Mégné N., Diallo K., Sembène P.M., Wade S. (2016). Physical Activity Level and Sedentary Behaviors among Public School Children in Dakar (Senegal) Measured by PAQ-C and Accelerometer: Preliminary Results. Int. J. Environ. Res. Public Health.

[32] Craig E., Bland R., Reilly J. (2012). Objectively measured physical activity levels of children and adolescents in rural South Africa: High volume of physical activity at low intensity. Appl. Physiol. Nutr. Metab..

[33] Bénéfice E., Garnier D., Ndiaye G. (2001). Assessment of physical activity among rural Senegalese adolescent girls: Influence of age, sexual maturation, and body composition. J. Adolesc. Health.

[34] Muthuri S.K., Wachira L.M., Vincent O., Onywera V.O., Tremblay M.S. (2014). Direct and self-reported measures of physical activity and sedentary by weight status in school-aged children: Results from ISCOLE-Kenya. Ann. Hum. Biol..

[35] Stevens J., Murray D.M., Baggett C.D., Elder J.P., Lohman T.G., Lytle L.A., Pate R.R., Pratt C.A., Treuth M.S., Webber L.S. (2007). Objectively assessed associations between physical activity and body composition in middle-school girls: The Trial of Activity for Adolescent Girls. Am. J. Epidemiol..

[36] Moliner-Urdiales D., Ortega F.B., Vicente-Rodriguez G., Rey-Lopez J.P., Gracia-Marco L., Widhalm K., Sjöström M., Moreno L.A., Castillo M.J., Ruiz J.R. (2010). Association of physical activity with muscular strength and fat-free mass in adolescents: The HELENA study. Eur. J. Appl. Physiol..

[37] Vicente-Rodriguez G., Dorado C., Ara I., Perez-Gomez J., Olmedillas H., Delgado-Guerra S., Calbet J.A. (2007). Artistic versus rhythmic gymnastics: Effects on bone and muscle mass in young girls. Int. J. Sports Med..

[38] Morelli C., Avolio E., Galluccio A., Caparello G., Manes E., Ferraro S., De Rose D., Santoro M., Barone I., Catalano S. (2020). Impact of Vigorous-Intensity Physical Activity on Body Composition Parameters, Lipid Profile Markers, and Irisin Levels in Adolescents: A Cross-Sectional Study. Nutrients.

[39] Collings P.J., Brage S., Ridgway C.L., Harvey N.C., Godfrey K.M., Inskip H.M., Cooper C., Wareham N.J., Ekelund U. (2013). Physical activity intensity, sedentary time, and body composition in preschoolers. Am. J. Clin. Nutr..

[40] Ebenegger V., Marques-Vidal P., Kriemler S., Nydegger A., Zahner L., Niederer I., Bürgi F., Puder J.J. (2012). Differences in aerobic fitness and lifestyle characteristics in preschoolers according to their weight status and sports club participation. Obes Facts.

[41] Bassett D.R., Toth L.P., LaMunion S.R., Crouter S.E. (2017). Step Counting: A Review of Measurement Considerations and Health-Related Applications. Sports Med..

[42] Wells J.C., Cole T.J., ALSPAC study steam (2002). Adjustment of fat-free mass and fat mass for height in children aged 8 y. Int. J. Obes Relat. Metab. Disord..

[43] Cole T.J., Fewtrell M.S., Prentice A. (2008). The fallacy of using percentage body fat as a measure of adiposity. Am. J. Clin. Nutr..

[44] Pereira-da-Silva L., Dias M.P., Dionísio E., Virella D., Alves M., Diamantino C., Alonso A., Cordeiro-Ferreira G. (2016). Fat mass index performs best in monitoring management of obesity in prepubertal children. J. Pediatr..

